# Supplementary material for: Complex‐centric proteome profiling by SEC‐SWATH‐MS
Source: Mol Syst Biol. 2019 Jan 14;15(1):e8438. doi: 10.15252/msb.20188438 (PMC6346213; doi:10.15252/msb.20188438)
Supplement: Supplementary file 8 — Dataset EV7 [file MSB-15-e8438-s008.zip › feature_plots_string/Q53GS9.pdf]

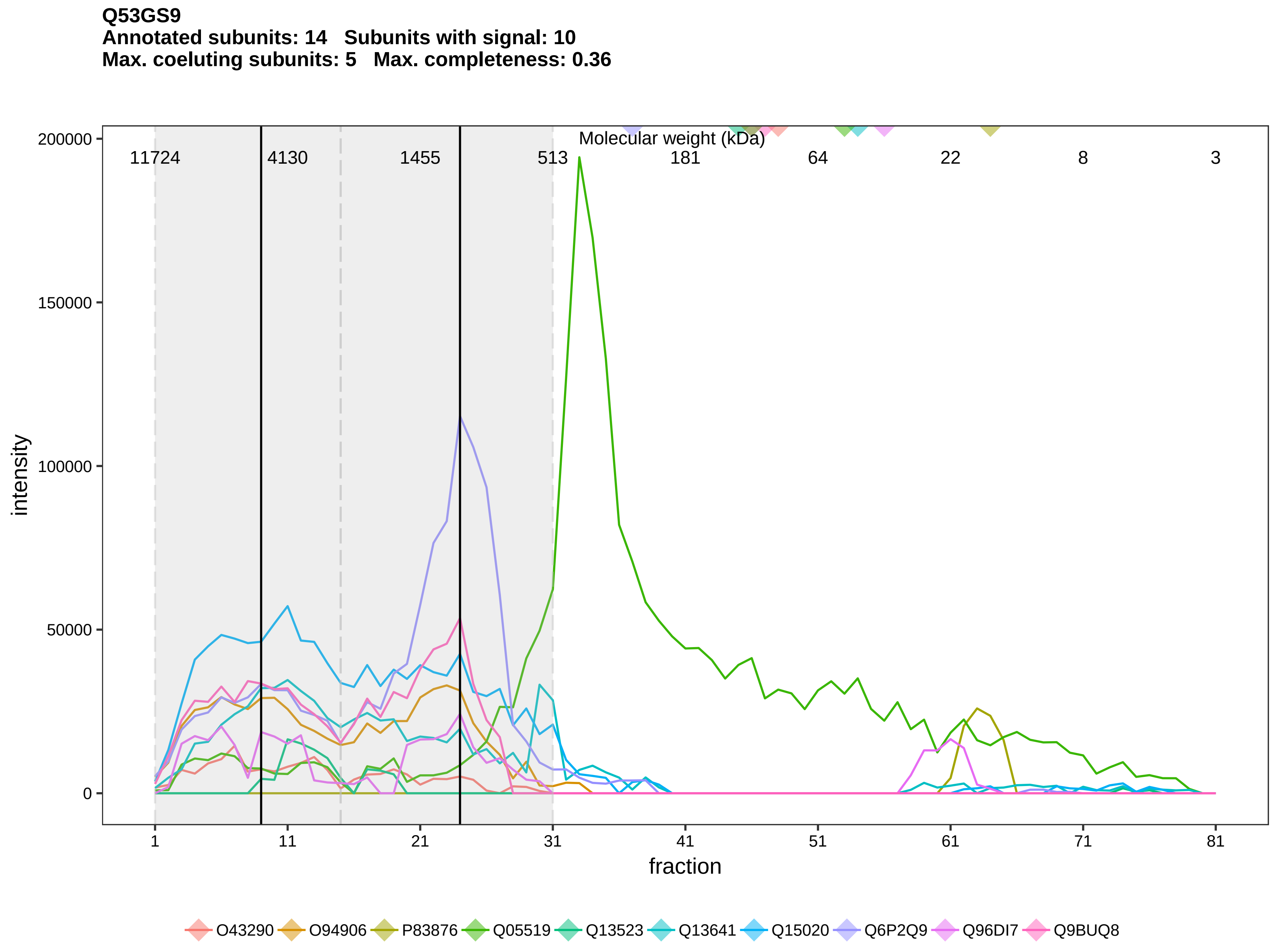

Q53GS9
Annotated subunits: 14 Subunits with signal: 10
Max. coeluting subunits: 5 Max. completeness: 0.36
Molecular weight (kDa)
11724
4130
1455
513
181
64
22
8
3
intensity
200000
150000
100000
50000
0
fraction
1
11
21
31
41
51
61
71
81
O43290
O94906
P83876
Q05519
Q13523
Q13641
Q15020
Q6P2Q9
Q96DI7
Q9BUQ8
